# Progress and perspectives of perioperative immunotherapy in non-small cell lung cancer

**DOI:** 10.3389/fonc.2023.1011810

**Published:** 2023-01-25

**Authors:** Yurong Peng, Zhuo Li, Yucheng Fu, Yue Pan, Yue Zeng, Junqi Liu, Chaoyue Xiao, Yingzhe Zhang, Yahui Su, Guoqing Li, Fang Wu

**Affiliations:** ^1^ Department of Oncology, the Second Xiangya Hospital, Central South University, Changsha, Hunan, China; ^2^ The Ophthalmologic Center of the Second Xiangya Hospital, Central South University, Changsha, Hunan, China; ^3^ XiangYa School of Public Health, Central South University, Changsha, Hunan, China

**Keywords:** perioperative period perioperative immunotherapy, neoadjuvant therapies, immunotherapy, neoadjuvant immune monotherapy, adjuvant therapy, biomarkers, NSCLC, lung cancer

## Abstract

Lung cancer is one of the leading causes of cancer-related death. Lung cancer mortality has decreased over the past decade, which is partly attributed to improved treatments. Curative surgery for patients with early-stage lung cancer is the standard of care, but not all surgical treatments have a good prognosis. Adjuvant and neoadjuvant chemotherapy are used to improve the prognosis of patients with resectable lung cancer. Immunotherapy, an epoch-defining treatment, has improved curative effects, prognosis, and tolerability compared with traditional and ordinary cytotoxic chemotherapy, providing new hope for patients with non-small cell lung cancer (NSCLC). Immunotherapy-related clinical trials have reported encouraging clinical outcomes in their exploration of different types of perioperative immunotherapy, from neoadjuvant immune checkpoint inhibitor (ICI) monotherapy, neoadjuvant immune-combination therapy (chemoimmunotherapy, immunotherapy plus antiangiogenic therapy, immunotherapy plus radiotherapy, or concurrent chemoradiotherapy), adjuvant immunotherapy, and neoadjuvant combined adjuvant immunotherapy. Phase 3 studies such as IMpower 010 and CheckMate 816 reported survival benefits of perioperative immunotherapy for operable patients. This review summarizes up-to-date clinical studies and analyzes the efficiency and feasibility of different neoadjuvant therapies and biomarkers to identify optimal types of perioperative immunotherapy for NSCLC.

## 1 Introduction

Lung cancer is the leading cause of death in men and women among neoplastic diseases globally. Nearly 85% of lung cancers are non-small cell lung cancer (NSCLC) (1). Surgical resection is the standard of practice for patients with operable early-stage and locally advanced NSCLC. However, 25%–70% of surgical patients (which varies by stage) eventually relapse despite complete resection, yielding a 5-year survival rate of 35%-65% (2). Distant metastasis is the most common form of lung cancer postoperative recurrence (3).

Perioperative treatments (adjuvant and neoadjuvant treatment) for NSCLC have the potential to improve disease outcomes. Three randomized controlled trials, namely, International Adjuvant Lung Cancer Trial (IALT), JBR.10, and Adjuvant Navelbine International Trialist Association (ANITA), and the pooled analysis in Lung Adjuvant Cisplatin Evaluation (LACE), studied the efficacy of adjuvant chemotherapy (4–6). Both adjuvant and neoadjuvant chemotherapy had an approximate 5-year survival benefit of 5% and did not significantly improve the time to local recurrence. However, additional regimens must be evaluated, and there is no phase III study of any perioperative targeted therapy.

Immune checkpoint inhibitors (ICIs) are mainstream of immunotherapy, with good clinical results in patients with advanced NSCLC (7–10). The PACIFIC study introduced immunotherapy to stage III NSCLC (11), reporting that patients with negative drive gene mutations can benefit from ICIs. The estimated 5-year overall survival (OS) of early-stage lung cancer was 23.2% for treatment-naive patients with pembrolizumab, and 15.5% *vs*. 16% for pembrolizumab versus nivolumab in previously treated patients (12).

**Figure 1:**
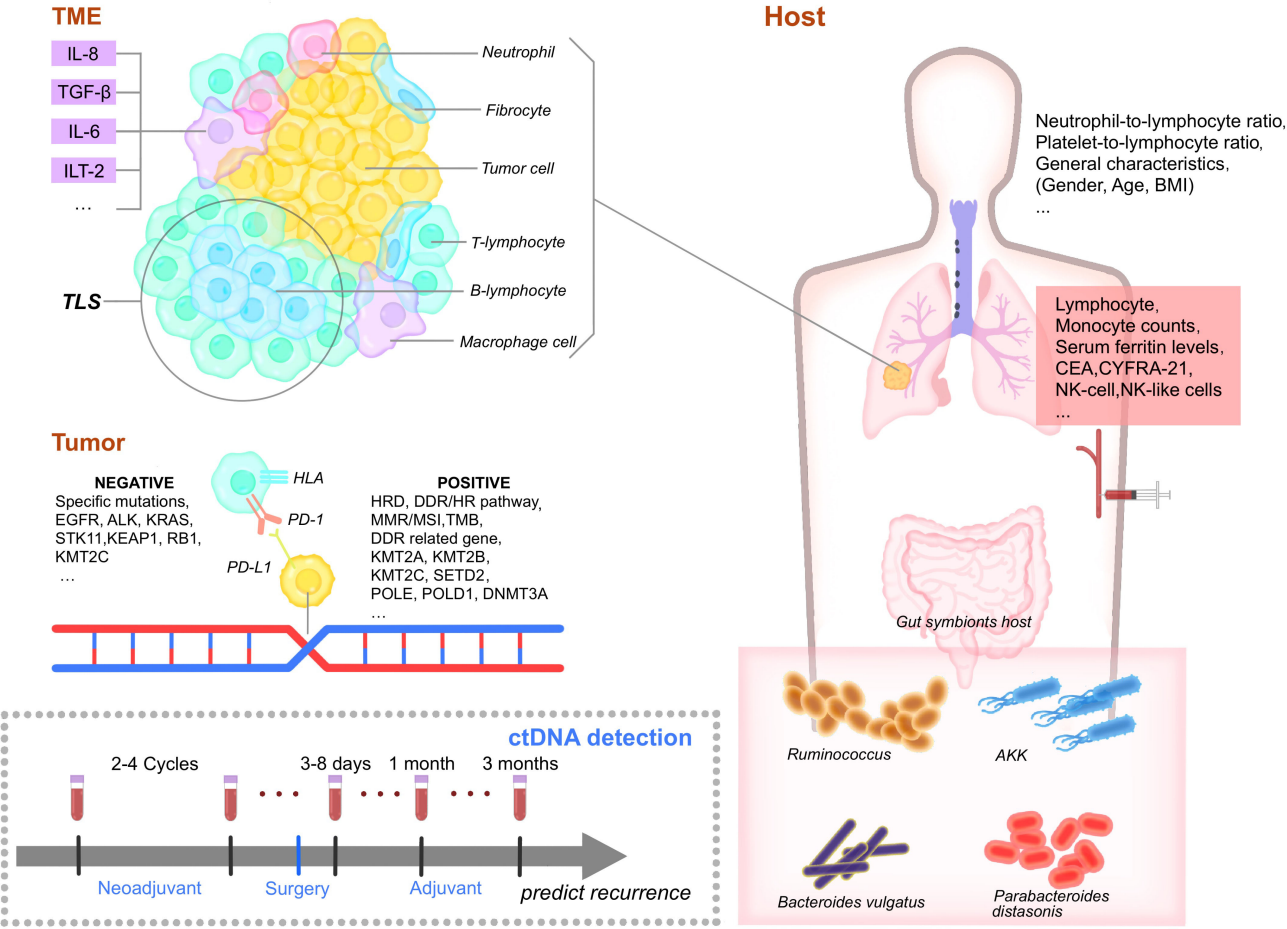
Biomarkers of perioperative immunotherapy. TME, Tumor microenvironment. IL-8, interleukin-8. TGF-β, Transforming growth factor. IL-6, interleukin-6. ILT-2, Ig-like transcript 2. TLS, Tertiary lymphoid structures. TIL, tumor-infiltrating lymphocytes. EGFR, epidermal growth factor receptor. HLA, human leukocyte antigen. PD-1, programmed cell death protein 1. PD-L1, Programmed cell death ligand 1. HRD, Homologous recombination deficiency. DDR, DNA-damage response/HR, homologous recombination pathway, MMR, mismatch repair. MSI, microsatellite instability. TMB, tumor mutation burden. KMT2A/B/C, Lysine methyltransferase 2A/B/C. POLE, polymerase epsilon. DNMT3A, DNA methyltransferases 3A. BMI, body mass index. CEA, carcinoembryonic antigen. NK, natural killer cells. AKK, Akkermansia muciniphila. NK, natural killer cells. CD4, cluster of differentiation 4. CD8+, cluster of differentiation 8. Treg, regulatory T cells. DC, dendritic cells. CTL, cytotoxic T lymphocyte.

Following the profound application of immunotherapy in NSCLC, there has been tremendous potential benefit to combining immunotherapy with surgery, as has been applied to some recent phase Ib/II and III clinical trials. The CheckMate159 trial was the first study that evaluated neoadjuvant immunotherapy, showing that neoadjuvant therapy with a single-drug programmed cell death protein 1 (PD-1) inhibitor (nivolumab) achieved a major pathological response (MPR) and pathological complete response (pCR) in 45% and 15% of participants, respectively (13). CheckMate-816 was the first phase III clinical trial to demonstrate the benefit of neoadjuvant immunotherapy (nivolumab) in resectable NSCLC patients, reporting a median event-free survival of nivolumab plus chemotherapy that was 10.8 months longer than with chemotherapy alone in addition to a pCR of 24.0% *vs*. 2.2%, respectively (14). This review incorporates the latest evidence to assess efficacy and feasibility of neoadjuvant immune monotherapy, immune-combination therapy, and biomarkers for neoadjuvant immunotherapy to identify the optimal perioperative immunotherapy for NSCLC (**Figure 1**).

## 2 Neoadjuvant immunotherapy

### 2.1 Neoadjuvant ICIs monotherapy

The earliest study of immunotherapy as perioperative neoadjuvant therapy for NSCLC was CheckMate 159, which enrolled 21 patients treated with nivolumab for two cycles before surgery. That trial opened a new era of perioperative immunotherapy, reporting an MPR rate of nivolumab neoadjuvant therapy of 45%, a 24-month relapse-free survival (RFS) of 70%, and a pCR of 10%. It also confirmed the clinical efficacy and safety of neoadjuvant ICI monotherapy (15). Despite initially promising results, the MPR rates reported by other studies that used single-agent neoadjuvant immunotherapy were not as encouraging. For instance, the LCMC3 study of neoadjuvant atezolizumab in patients with resectable stage IB-IIIB NSCLC with epidermal growth factor receptor (EGFR) or anaplastic lymphoma kinase (ALK) genetic aberrations attained a 21% of MPR at the time of resection and a pCR of 7%, while the use of atezolizumab in another neoadjuvant study yielded an MPR rate of only 13% and a pCR of 7%. These data suggest that atezolizumab has a survival advantage compared with historical outcomes, but more convincing evidence is needed to validate its curative effect.

When Nivolumab single-agent treatment was evaluated in the NEOSTAR study, the MPR rate was only 17% and the pCR was 9% (16). Moreover, the use of sintilimab in 49 patients in ChiCTR-OIC-17013726 and durvalumab in 46 patients in IONESCO as single-agent treatment with an ICI in the neoadjuvant setting reported MPR rates of 40% and 17% and pCRs of 16% and 7%, respectively (17–19). The TOP1501 study showed that pembrolizumab was efficacious and well tolerated as neoadjuvant therapy in 30 patients (20). These results indicate that different monotherapies with anti-PD-L1 agents are being attempted, but more clinical studies are required to establish the ability for neoadjuvant immunotherapy to improve the survival of patients with early-stage NSCLC (**Table 1**).

**Table 1:** Preoperative phase I or II neoadjuvant immunotherapy in operable NSCLC.

	Clinical trial	Phase	Stage	Intervention used	Estimated sample size	Primary endpoints	Secondary endpoints	Estimated completion date
Neoadjuvant ICIs monotherapy	ChiCTR-OIC-17013726	I	IA-IIIB	Sintilimab 2C + S	40	Safety	ORR,MPR,DFS	05/2020
	MK3475-223 (NCT02938624)	I	I-II	Pembrolizumab 1C/2C+ S	28	Toxicity, MPR	mOS, mTR	04/2021
	CheckMate 159 (NCTO2259621)	II	l-IIIA	Nivolumab 2C + S	45	Safety	PR, RdR	01/2023
	IONESCO (NCT03030131)	II	IB-II	Durvalumab 3C + S	81	R0 resection	RR, DFS,OS	08/2019
	Columbia University (NCT02716038)	II	IB-IIIA	Atezolizumab 4C + S	30	MPR	NA	12/2021
	PRICNEPS (NCT02994576)	II	IB-IIIA	Atezolizumab 1C + S	60	Toxicity	NA	12/2022
	NEOMUN (NCT03197467)	II	II-IIIA	Pembrolizumab 2C +S	30	AEs	DFS, OS	10/2023
	LCMC3 (NCT02927301)	II	IB-IIIA	Atezolizumab 2C+ S	180	MPR	ORR	05/2025
	TOP1501 (NCT02818920)	II	IB-IIIA	Pembrolizumab+ S	35	SFR	ORR, DFS	03/2026
Neoadjuvant ICIs immune-combination therapy	NeoCOAST(NCT03794544)	II	I-IIIA	Durvalumab ± Oleclumab (MEDI9447) or Monalizumab (IPH2201) or Danvatirsen+ S	160	MPR	pCR	01/2021
	CANOPY-N (NCT03968419)	II	IB-IIIA	Canakinumab + Pembrolizumab/Canakinumab/Pembrolizumab	88	MPR	ORR	08/2022
	NADIM (NCT03081689)	II	I-IIIA	Chemotherapy + Nivolumab *vs*. Chemotherapy+ S	46	PFS	OS, Toxicity	06/2023
	SAKK 16/14 (NCT02572843)	II	IIIA (N2)	Durvalumab 2C + Chemotherapy 3C+ S	68	EFS	OS, OR, pCR	12/2024
	NEOSTAR (NCT03158129)	II	l-IIIA	Nivolumab ± Ipilimumab or Chemotherapy	88	MPR	RFS	07/2022
	TOP1201 (NCT01820754)	II	IB-IIIA	Chemotherapy 1C + (Ipilimumab + Chemotherapy) 2C + S	24	CTCs	Toxicity, mDFS	04/2018
	EAST ENERGY (NCT04040361)	II	IB-IIIA	Pembrolizumab + Ramucirumab + S	24	MPR	Safety, pCR, OS, ORR	11/2025

NSCLC, non-small cell lung cancer; C: cycle; S: surgery; y: year; ORR, objective response rate; MPR, major pathological response; DFS, disease-free survival; mOS, median overall survival; mTR, median time-to-recurrence; PR, pathological response; RdR, radiographic response; RR, response rate; R0 resection; OS, overall survival; NA, not mentioned; patient percentage of surgical resection R0 after a maximum of three cycles of immune therapy; AEs, adverse events; CTCs, circulating T cells; EFS, event-free survival; PFS: progression-free survival; pCR, pathological complete response; SFR, surgical feasibility rate; RFS, recurrence-free survival; R0, resection.

To summarize, the MPR ratio of perioperative ICI monotherapy of 14%–45% is significantly higher than that of neoadjuvant chemotherapy with cisplatin (MPR between 10% and 15%), but the clinical benefit rate of these treatments was not as high.

### 2.2 Neoadjuvant immune-combination therapy

It is generally felt that neoadjuvant immune-combination therapy is a great step forward in perioperative immunotherapy. Versatile forms of combination therapy, such as neoadjuvant chemoimmunotherapy, neoadjuvant immunotherapy plus antiangiogenic therapy, neoadjuvant dual immunotherapy, neoadjuvant immunotherapy plus radiotherapy or concurrent chemoradiotherapy, and neoadjuvant immunotherapy plus chemoradiotherapy, are currently being investigated (**Tables 1**, **2**).

**Table 2:** Ongoing clinical trials of neoadjuvant immunotherapy plus radiotherapy in operable NSCLC.

Clinical trial	Phase	Stage	Drugs		Intervention used	Estimated sample size	Primary endpoints	Secondary endpoints	Estimated completion date
NCT04287894	I	II–III	Durvalumab		Durvalumab 2C+ Chemotherapy + Radiotherapy + S + Durvalumab	34	Safety	DFS, OS, DCR	01/2021
NCT05157542	I	III	Durvalumab		Durvalumab 2C + Chemotherapy + Radiotherapy+ S	9	Safety, AEs, SAEs	ORR, EFS, MPR	06/2023
NCT02987998	I	IIIA	Pembrolizumab		Pembrolizumab + Chemotherapy + Radiotherapy + S + Pembrolizumab	9	Safety	PFS, ORR	01/2024
NCT03237377	II	IIIA	Durvalumab or Tremelimumab(CTLA-4)		Durvalumab 3C + Radiotherapy + S *vs*. Durvalumab 3C + Tremelimumab(CTLA-4)+ Radiotherapy + S	32	Safety, feasibility	SMM, PR rate	09/2021
NCT03217071	II	I-IIIA	Pembrolizumab		Pembrolizumab 2C + S *vs*. Pembrolizumab 2C + Radiotherapy(SRT) + S	40	number of infiltrating CD3+ T cells/μm2	AEs, OS, RFS	12/2021
NCT02904954	II	IB-IIIA	Durvalumab		Durvalumab 2C + S + Durvalumab 1y *vs*. Durvalumab 2C + Radiotherapy + S + Durvalumab 1y	60	MPR	DFS, ORR	04/2022
NCT04085250	II	III	Nivolumab		Nivolumab 2C + Chemotherapy +Radiotherapy + S + Nivolumab 1y	264	PFS	OS, ORR, AEs	11/2023
NCT04933903	II	IB-III	ipilimumab +Nivolumab		Ipilimumab + Nivolumab + SBRT+ S	25	MPR, pCR	AEs	01/2024
NCT03110978	II	I-IIA	Radiotherapy (SBRT) + Nivolumab *vs*. Radiotherapy		SBRT *vs*. SBRT+ Nivolumab 3C	140	EFS, secondary malignancy, and death	OS, AEs	06/2022
NCT04245514	II	T1-4>7 N2	Durvalumab		Durvalumab 1C + Chemotherapy 3C+Radiotherapy+ S + Durvalumab 13C	90	EFS	RFS, OS, pCR, MPR	03/2025

NSCLC, non-small cell lung cancer; C: cycle; S: surgery; y: year; DCR, 1-year disease control rate; CTLA-4, immunoglobulin-related receptors that are responsible for various aspects of T-cell immune regulation; SRT, stereotactic radiation therapy; SBRT, stereotactic body radiation therapy; AEs, adverse events; SAEs, serious adverse events; ORR, objective response rate; RFS, relapse-free survival; EFS, event-free survival; DFS, disease-free survival; SMM, surgical morbidity and mortality; PR rate, pathological response; PFS, progression-free survival; MPR, major pathological response; pCR, pathological complete response; EFS, event-free survival; OS, overall survival; T1–4>7 N2: i.e., T1–3 N2 or T4 N2 but T4 only allowed if due to size >7cm, not allowed if due to invasion or nodule in different ipsilateral lobe.

#### 2.2.1 Neoadjuvant chemoimmunotherapy

Neoadjuvant chemoimmunotherapy accounts for the majority of neoadjuvant immunotherapy clinical trials and generally reports an improved pathological response, with a higher pCR and MPR compared with single-agent neoadjuvant immunotherapy, thereby prolonging OS.

There may be some synergy between neoadjuvant immunotherapy and chemotherapy. Chemotherapy can induce tumor cell gene mutations, thereby producing new epitopes that can in turn enhance tumor immunogenicity and improve the efficacy of immunotherapy (21).

In a phase II trial of 30 patients with stage IB–IIIA NSCLC, neoadjuvant atezolizumab combined with chemotherapy achieved MPR and pCR rates of 57% (17/30) and 33% (10/30), respectively (22). Of the 55 patients with stage IIIA NSCLC in the SAKK 16/14 study who underwent surgical resection followed by the treatment of cisplatin or docetaxel followed by durvalumab, the MPR rate was 62% (34/55), the pCR rate was 18% (10/55), and the 1-year event-free survival (EFS) rate reached 73.3% (23). Toripalimab plus platinum-based doublet chemotherapy for patients with stage III NSCLC yielded a high MPR rate of 66.7% and a pCR rate of 50% (24). Although neoadjuvant chemoimmunotherapy had good therapeutic efficacy, treatment-related adverse events are worth mentioning. A single-arm trial of 21 patients who underwent two cycles of neoadjuvant nivolumab every 2 weeks before surgery was associated with few side effects, no delay in surgery, and an MPR of 45% (9/20) (15).

The NADIM study with nivolumab plus paclitaxel and carboplatin achieved strong clinical results, with an MPR of 83% (34/41), a pCR of 63% (26/41), a 2-year progression-free survival (PFS) rate of 77.1%, and a 2-year OS of 89.9%. However, 93% (43/46) of the patients had treatment-related adverse events, 30% (14/46) of which were in grade ≥ 3. However, none of the adverse events were associated with surgery delays or treatment-related deaths (25). The SAKK 16/14 study also reported grade ≥ 3 adverse events in 59 (88%) patients, including two fatal adverse events that were judged to not be treatment related (23).

The first phase III study to evaluate neoadjuvant chemoimmunotherapy, CheckMate-816, with neoadjuvant nivolumab plus chemotherapy achieved an approximately 10-fold meaningful increase, with a pCR of 24% (95% CI, 18.0–31.0) compared with 2.2% (95% CI, 0.6–5.6) among patients treated with chemotherapy alone. The EFS of the experimental group was 31.6 months compared with 20.8 months in the single-agent chemotherapy group, representing a significant improvement. The US Food and Drug Administration recently approved CheckMate-816’s regimen, which could represent a new standard of care for NSCLC patients with tumors ≥4 cm or who are node positive (14). Several other relevant phase III clinical trials are currently underway, such as the AEGEAN study, which focuses on durvalumab combined with chemotherapy, and their results are highly anticipated (26).

The treatment intervals between cycles varied among all of the above study designs. The interval between toripalimab, nivolumab, tislelizumab, durvalumab, and camrelizumab cycles were generally 2 weeks before surgery, while for pembrolizumab and atezolizumab, it was 3 weeks. As a result of comprehensive consideration of various factors, most studies chose two to four immune treatment cycles to ensure its efficacy and patient compliance, but more clinical evidence is required to identify the optimal medication regimen (27–29).

#### 2.2.2 Neoadjuvant immunotherapy plus antiangiogenic therapy

Previous studies have demonstrated that antiangiogenic drugs (Endostar) combined with chemotherapy in patients treated with neoadjuvant therapy can increase therapeutic efficacy without increasing adverse effects in stage IIIA-N2 NSCLC patients (30). Several recent clinical studies evaluated neoadjuvant immunotherapy combined with antiangiogenic therapy. The phase 2 study NCT04040361 administered two cycles of pembrolizumab and ramucirumab before surgery, with MPR defined as the primary outcome measure. Anlotinib was combined with pembrolizumab in a neoadjuvant study (NCT04762030). Additional future trials may evaluate other ICIs along with various antiangiogenic drugs. The optimal combination of these therapies, the ideal target population, and therapy choice in the setting of disease progression require further study.

#### 2.2.3 Neoadjuvant dual immunotherapy

Cascone et al. sought to examine the efficacy of neoadjuvant immune-immune therapy through the NeoSTAR, which evaluated anti-PD-1 plus anti-CTLA-4 in the treatment of early-stage NSCLC. Compared with nivolumab, nivolumab plus ipilimumab had higher MPR (22% *vs*. 38%) and pCR rates (10% *vs*. 38%), suggesting that dual immunotherapy has significant potential during the perioperative period in patients with operable NSCLC (16). The recent NeoCOAST study paired neoadjuvant durvalumab with three investigational drugs, namely, oleclumab, monalizumab, and danvatirsen, reporting that combination strategies may boost the programmed cell death ligand 1 (PD-L1) inhibitor durvalumab’s neoadjuvant efficacy, resulting in MPR rates of 19%, 30%, and 31.3% with oleclumab, monalizumab, and danvatirsen, respectively, compared with durvalumab monotherapy (11.1%) (31). Ongoing clinical studies are evaluating the efficacy and safety of two cycles of neoadjuvant durvalumab immunotherapy plus ramucirumab (anti-angiogenic) (NCT04040361), durvalumab combined with FL-101 (anti-IL-1β, NCT04758949), oleclumab (anti-CD73) plus chemotherapy, and monalizumab (anti-NKG2A) plus chemotherapy (NCT05061550).

#### 2.2.4 Neoadjuvant immunotherapy plus radiotherapy or concurrent chemoradiotherapy

Radiotherapy is a standard treatment for many tumors. Radiotherapy cannot only kill the inhibitory stromal cells, indirectly improving the body’s anti-tumor activity, but also induce immunogenic cell death and expose the surface of calpain cells. The release of immunostimulatory components such as High Mobility Group Box 1 (HMGB1) and adenosine triphosphate (ATP) activates dendritic cells and effector T cells, which in turn increase the body’s anti-tumor abilities. Radiotherapy can also induce the expression of various proinflammatory cytokines, such as interleukin-1β (IL-1β) and tumor necrosis factor α (TNF α), which can induce the tumor’s inflammatory microenvironment and increase its immune tumor necrosis factor (32, 33).

Neoadjuvant immunotherapy plus radiotherapy achieved positive results in recent clinical trials. A study of neoadjuvant chemoradiation and durvalumab in patients with potentially resectable phase III NSCLC tumors reported a 77.8% MPR rate (14/18, 95% CI, 54.3%–91.5%) and a 38.9% pCR rate (7/18, 95% CI, 20.2%–61.5%). Seventy-five percent (18/24) of patients underwent surgery after neoadjuvant therapy (34). Neoadjuvant durvalumab with or without stereotactic body radiotherapy (SBRT) also achieved better outcomes, with MPR observed in 16 of 30 patients (53.3%) in the durvalumab plus radiotherapy group *vs*. 2 of 30 patients (6.7%) in the durvalumab monotherapy group (35). These results suggest that neoadjuvant immunotherapy plus radiotherapy or chemoradiotherapy are more effective than neoadjuvant ICI monotherapy. However, this concept is being further evaluated (**Table 2**).

## 3 Adjuvant immunotherapy

Postoperative adjuvant therapy can eliminate undetectable residual “micrometastatic” tumor cells that may exist in lymph nodes, blood vessels, or lymphatic vessels, delaying or reducing postoperative recurrence and metastasis, prolonging PFS and OS, and improving patient quality of life. Adjuvant immunotherapy for perioperative patients includes adjuvant immune monotherapy and combination therapy (36).

With respect to adjuvant chemo-immunotherapy therapy, atezolizumab after adjuvant chemotherapy is a promising treatment option for patients with resectable early-stage NSCLC. The Impower010 study, the first to incorporate immunity into early-stage lung cancer treatment (atezolizumab), achieved a 34% improvement in disease-free survival (DFS) in stage II–IIIA patients with PD-L1≥1%, an improvement also supported by the OS interim analysis of atezolizumab at the 2022 American Society of Clinical Oncology Meeting (37). Based on the success of Impower010 study and the approval of the Food and Drug Administration (FDA), postoperative auxiliary immunotherapy became the standard guidance (38).

Patients with stage II–IIIA PD-L1-positive tumors and PD-L1 tumor proportion scores (TPS) ≥ 50% are considered the key targets for immune adjuvant therapy. Keynote-091, a randomized, triple-blinded, phase III trial, enrolled a total of 1,177 patients who were randomized 1:1 to receive either pembrolizumab or placebo. The study’s dual primary end points were DFS in all-comers and PD-L1 TPS ≥50% groups. It was announced at the 2022 European Society for Medical Oncology virtual plenary meeting that the study had reached one of the two primary endpoints (39). Pembrolizumab significantly improved the cohort’s DFS regardless of the PD-L1 expression level (53.6 *vs*. 42.0 months; HR, 0.76; 95% CI, 0.63–0.91; p = 0.0014). These results suggest that immunotherapy is an effective adjuvant treatment for NSCLC and could significantly prolong the postoperative DFS of NSCLC patients. It also laid the foundation for further studies seeking to evaluate adjuvant immunotherapy after surgery.

Other phase III clinical trials that are currently underway are evaluating the efficacy of adjuvant immunotherapy in patients with resected stage IB–IIIA NSCLC (**Table 3**).

**Table 3:** Ongoing phase III clinical trials of neoadjuvant and adjuvant immunotherapy in operable NSCLC.

	Clinical trial	Stage	Drugs	Intervention used	Estimated sample size	Phase	Primary endpoints	Estimated completion date
Adjuvant Immunotherapy	IMpower010 (NCT02486718)	IB (tumors ≥4 cm) to IIIA	Atezolizumab	S+ Atezolizumab + Chemotherapy/Chemotherapy alone	1280	III	DFS	12/2027
	PEARLS/KEYNOTE-091 (NCT02504372)	IB (tumors ≥4 cm) to IIIA	Pembrolizumab (MK-3475)	S+ Pembrolizumab 1y/placebo	1177	III	DFS	02/2024
	ANVIL (NCT02595944)	IB (tumors ≥4 cm) to IIIA	Nivolumab	S+ Nivolumab 1y/placebo	903	III	DFS	07/2024
	IFCT-1401 (NCT02273375)	IB (tumours ≥4 cm) to IIIA	MEDI4736	S+ MEDI4736 1y/placebo	1415	III	DFS	01/2024
	MERMAID 1 (NCT04385368)	II-III	Durvalumab	Durvalumab + SoC Chemotherapy/placebo	86	III	DFS	12/2026/
	MERMAID 2 (NCT04642469)	II-III	Durvalumab	S+durvalumab(1y)/placebo	284	III	DFS	10/2027
Neoadjuvant Immunotherapy +Chemotherapy	AEGEAN(NCT03800134)	IIA -IIIB	Durvalumab	Durvalumab+Chemotherapy + S	800	III	MPR	01/2024
	CheckMate 816(NCT02998528)	IB-IIIA	Nivolumab	Nivolumab+Chemotherapy/Nivolumab+Ipilimumab/Chemotherapy + S	350	III	EFS, pCR	11/2028
Neoadjuvant + Adjuvant Immunotherapy+Chemotherapy	KEYNOTE-671(NCT03425643)	IIB-IIIA	Pembrolizumab	Pembrolizumab+Chemotherapy S + Pembrolizumab	786	III	EFS, OS	06/2026
	CheckMate 77T(NCT04025879)	II-IIIB (T3N2)	Nivolumab	Nivolumab+Chemotherapy S + Nivolumab	452	III	EFS	09/2024
	IMpower 030(NCT03456063)	II-IIIB (T3N2)	Atezolizumab	Atezolizumab+Chemotherapy S + Atezolizumab	450	III	MPR, EFS	11/2024
	RATIONALE 315(NCT04379635)	II-IIIA	Tislelizumab	Tislelizumab+Chemotherapy S + Tislelizumab	380	III	MPR, EFS	02/2021
	JS001-029 (NCT04158440)	IIIA	Toripalimab	Toripalimab+Chemotherapy S + Toripalimab	406	III	MPR, EFS	10/2024
	NCT05116462	IIB-IIIB	Sintilimab	Sintilimab+Chemotherapy S + Sintilimab	800	III	EFS, pCR	06/2026

NSCLC, non-small cell lung cancer; C, cycle; S, surgery; y, year; SoC, Standard of care DFS, disease-free survival; MPR, major pathologic response; EFS, event-free survival; pCR, pathologic complete response; OS, overall survival.

## 4 Neoadjuvant and adjuvant immunotherapy

Prior to resection, the patient’s tumor is large and neoantigen abundant and their immune system is relatively intact. Anti-PD-1 therapy at this stage can induce the expansion of mutation-associated neoantigen-specific T-cell clones in the peripheral blood (13). It can also fully enhance the activity of anti-tumor immune T cells *in vivo*. However, the surgery can contribute to change in cytokines, growth factors, and immune cells because of inflammation and neuroendocrine and postoperative complications, resulting in immunosuppression (40).

Adjuvant therapy will be primarily used in patients with resectable stage l and II NSCLC, but neoadjuvant therapy is more preferred for patients with stage IIIA to IIIC disease (41).

Moreover, the interval between neoadjuvant therapy and surgery is of great importance. A preclinical study in mouse models of spontaneously metastatic mammary cancer reported that a short duration (4–5 days) between the first administration of neoadjuvant immunotherapy and resection of the primary tumor was necessary to achieve optimal efficacy. The authors also found that changes in the immune microenvironment, including differences in the proportion of tumor-specific T cells and their ability to produce interferon gamma (IFNγ), influence operative timing (42).

NADIM, NADIM II, and CheckMate 816 all reported a consistent and reproducible improvement in the rate of pathological response in patients treated with neoadjuvant immunotherapy in combination with chemotherapy (14, 25). NADIM II demonstrated superior pCR in patients with resectable stage IIIA NSCLC treated with chemotherapy combined with immunotherapy. Patients were only included in the adjuvant immune treatment (nivolumab) cohort if they were R0 and received their first drug administration between the third to eighth week after surgery and over a 6-month period. Results showed that neoadjuvant nivolumab plus chemotherapy significantly increased pCR compared with chemotherapy in the intent-to-treat patients (ITT, 36.2% *vs*. 6.8%) and reported an improved MPR rate (52% *vs*. 14%) and ORR (74% *vs*. 48%) versus chemotherapy alone (43).

Neoadjuvant and adjuvant immunotherapy combined with chemotherapy has been studied clinically. The majority of these studies evaluated two to four cycles of neoadjuvant treatment and 1 year for effective adjuvant therapy. Tislelizumab was evaluated for 8 cycles and atezolizumab 16 cycles. Ongoing phase III trials are exploring the safety and feasibility of neoadjuvant immunotherapy combined with chemotherapy for NSCLC. Different drugs (pembrolizumab, nivolumab, atezolizumab, tislelizumab, toripalimab, and sintilimab) were evaluated in combination with chemotherapy versus chemotherapy alone, mostly using EFS as the primary endpoint (**Table 3**).

## 5 Biomarkers for perioperative immunotherapy

Immunotherapy biomarkers for patients with resectable NSCLC can be roughly divided into four groups: tumor-cell-associated biomarkers, tumor-microenvironment (TME)-associated biomarkers, host-associated biomarkers, and blood cell and liquid biopsy-related biomarkers.

### 5.1 Tumor-cell-associated biomarkers

Tumor biomarkers for NSCLC are substances present in or produced by the tumor itself or the host microenvironment in response to tumorigenesis and progression. The tumor-cell-related biomarkers of interest to perioperative immunotherapy include PD-L1, TMB, the DNA–damage response (DDR) pathway, the homology-dependent recombination (HR) pathway, homologous recombination deficiency (HRD), specific genetic mutations (e.g., the interferon gamma pathway, KRAS, and STK11 mutations), and neo-antigens. All of the above may be related to the efficacy of perioperative immunotherapy (44).

#### 5.1.1 Programmed cell death ligand 1

PD-L1 antibody blocking immune checkpoints have revolutionized the treatment of advanced NSCLC (15). CheckMate159 study was the first to report a correlation between PD-L1 expression and MPR/RFS in immune single-agent neoadjuvant therapy. PD-L1 expression was associated with pathological remission, and PD-L1-positive tumors trended towards improved RFS. LCMC3 study reported on the pathological and imaging response to different levels of PD-L1 expression as secondary endpoints, finding that high levels of PD-L1 expression was associated with MPR from neoadjuvant immunotherapy in patients with early stage NSCLC (45). A meta-analysis that studied 10 neoadjuvant immunotherapy studies and included a total of 461 NSCLC patients associated high levels of PD-L1 expression with an improved pathological response, noting 50% PD-L1 as a stronger predictive cutoff than 1% expression. A report presented at the 2022 ASCO meeting reported that neoadjuvant nivolumab treatment of tumors with high PD-L1 expression may predict long-term response. However, larger prospective studies are required (46).

PD-L1 expression was also found meaningful when considering adjuvant immunotherapy. Impower010 showed that patients with PD-L1 expression≥xprhad a greater DFS benefit, especially in patients high expression levels (38).

There is no consensus on the use of PD-L1 to estimate the efficacy of neoadjuvant treatment. CheckMate-816 reported that patients with ≥1% PD-L1 expression had a considerably higher treatment benefit than those with <1% expression (14). Similarly, NADIM II reported pCR rates of patients with PD-L1 expression 1%-49% or TPS ≥ 50% of 41.7% and 61.1%, respectively, compared with 15% in patients with PD-L1 expression <1%. Moreover, patients with high levels of PD-L1 expression and TPS ≥ 1% were more likely to achieve pCR after nivolumab plus chemotherapy (43). ChiCTR-OIC-17013726’s 3 year results found that patients with PD-L1 ≥ 1% had more favorable clinical outcomes than other subgroups (HR, 0.275; 95% CI, 0.078–0.976) (19). Another study of neoadjuvant durvalumab alone or combined with SBRT reported that MPR was achieved independent of PD-L1 tumor status after adjusting for PD-L1 baseline expression as assessed with immunohistochemistry (IHC). Furthermore, no significant changes in PD-L1 expression were observed when comparing pretreatment and surgical resection tumor specimens in both trial groups and between patients with and without MPR (35). A further study showed that there was no significant association between PD-L1 expression and PFS (47).

#### 5.1.2 Tumor mutation burden

TMB is defined as the number of somatic mutations per million bases in the coding region of the tumor genome. In patients with advanced NSCLC being treated with immunotherapy, TMB is closely related to the efficacy and prognosis of immune checkpoint inhibitors. This is not as well shown in patients with early NSCLC who receive perioperative immunotherapy, with current studies still in the exploratory stage. However, it is generally accepted that TMB is closely related to the efficacy and prognosis of ICIs (48).

A study of neoadjuvant nivolumab for patients with stage I–IIIA NSCLC reported an MPR rate of 45%, with 311 patients with MPR and 74 patients without, which was statistically significant (p = 0.01). Furthermore, it showed that there was a significant correlation between pathological response and the pretreatment tumor mutational burden (15). A recent meta-analysis suggests that TMB may be associated with better pathological response to neoadjuvant immunotherapy, and although there are different neoadjuvant and adjuvant regimens and different TMB detection methods [next-generation sequencing (NGS) and whole-exome sequencing (WES)], high TMB is always associated with high MPR and pCR rates (49). In LCMC3, patients with high TMB values tended to have a better pathological response, and a high TMB immune response was associated with better PFS. This indicates that TMB is a potential predictive biomarker for MPR during neoadjuvant immunotherapy. After it was found in KENOTE-158 that patients with a high TMB performed significantly better than those with a low TMB, pembrolizumab monotherapy was approved by the FDA for patients with a high TMB, defined as ≥10 mutations/Mb, those with disease progression after previous treatments, and for patients with solid metastatic tumors. However, in another study on neoadjuvant immunotherapies, the MPR of nivolumab combined with ipilimumab was only 33%, indicating that the pathological response was not associated with TMB (50). The predictive value of TMB for neoadjuvant immunotherapy therefore requires further study.

#### 5.1.3 Homologous recombination deficiency

HRD are biomarkers that may be highly predictive of the therapeutic outcomes of neoadjuvant immunotherapy in NSCLC patients. Mutations of tumor suppressor genes in the DNA damage repair (DDR) and homology-dependent recombination (HR) pathways were more common in MPR patients, suggesting that better responders were likely to have HRD events. Moreover, HR pathway mutations were associated with better responding immunotherapy patients regardless of the treatment regimen and clinicopathological characteristics. It has also been observed that patients on immunotherapy with HR mutations have higher a TMB and longer survival in addition to a substantial number HR pathway alternations in multiracial treatment-free samples (51, 52).

Mismatch repair (MMR) is one of the multiple pathways that compose the DDR system. The proteins of MMR are related to apoptosis, indicating that the TME can indirectly promote the survival of tumor cells by inhibiting some DDR pathways. Defects in MMR genes that result in microsatellite instability-high (MSI-H) status result in the accumulation of mutations and the production of neoantigens, which can enhance the anti-cancer immune response (53). DDR-related genes such as KMT2A, KMT2B, KMT2C, SETD2, POLE, POLD1, and DNMT3A may also be predictive biomarkers for immunotherapy outcomes in patients with resectable NSCLC (52).

#### 5.1.4 Oncogenic driver mutations

NSCLC patients with mutations in major driver genes, such as EGFR and ALK, are excluded or represent a very small proportion of the cohorts used in most immunotherapy clinical studies. This indicates that conventional immunotherapy is not recommended for driver-gene-positive NSCLC patients, especially during neoadjuvant period (27).

KEYNOTE-010, a phase III randomized clinical trial comparing pembrolizumab to docetaxel, found in its subgroup analysis of EGFR-mutated NSCLC patients that pembrolizumab did not improve OS compared with docetaxel. Only a modest proportion of patients benefited from targeted treatment, and while no additional benefits from ICI therapy were observed, drug toxicity and side effects were report (54). Neoadjuvant single-agent immunotherapy in patients with potentially negative factors, such as EGFR-sensitive mutations/ALK fusions, should be used with caution. EGFR/ALK mutations are promising predictive biomarkers.

In the NADIM study, patients with driver gene EGFR/STK11/KEAP1/RB1 mutations had shorter PFS than wild-type patients, suggesting that patients with these mutations are less likely to benefit from neoadjuvant immune-chemotherapy (25).

With respect to STK11 mutation status, KEYNOTE-042 showed that patients who received pembrolizumab monotherapy had better PFS and OS than those who received standard chemotherapy. It is unclear if a STK11 mutation is a prognostic or predictive factor in patients with NSCLC who are receiving PD-1/PD-L1 inhibitor therapy (10).

### 5.2 Tumor-microenvironment-associated biomarkers

The overall immune microenvironment can be considered a biomarker of immunotherapy efficacy. A growing body of evidence suggests that microbiome is associated with ICIs and could certainly influence the efficacy of neoadjuvant immunotherapy. Tumor-microenvironment-associated biomarkers include tumor-infiltrating immune cells and immune status scores (55). The former consists of immune cells with specific phenotypes (e.g., cluster of differentiation 4+, CD4+ T cells, cluster of differentiation 8+, CD8+ T cells, and FOXP3+ T cells) and the diversity of the immune repertoire (e.g., T-cell receptor library).

Early studies have shown that massive infiltration of CD4+ and CD8+ T cells is associated with better tumor survival and prognosis (56). In the excised specimens of patients who achieved pCR from immune-neoadjuvant therapy, a large number of infiltrated CD8+ T cells, PD-1+ lymphocytes, CD68+ macrophages, FoxP3+ regulatory T cells, and tertiary lymphocytes were observed in the visual field. Higher levels of CD3+ tumor-infiltrating lymphocytes (TILs) and tissue-resident memory T cells were also seen in surgical specimens (50). Similarly, CD3+ and PD-1+ T cells were increased in patients with MPR in LCMC3 (NCT02927301) (45). Findings from CheckMate159, which evaluated nivolumab single-agent neoadjuvant therapy, suggested that T-cell enrichment could be a potential biomarker, as patients who achieved MPR after receiving neoadjuvant therapy with nivolumab had higher levels of CD8+ T-cell infiltration after treatment compared with before treatment. This suggests that PD-1 inhibitors may enhance anti-tumor T-cell activation (13). In another study cohort, high CD8+ TILs IHC expression was associated with better OS (9.4 *vs*. 5.6 months) (47). It is important to note that in the resected specimens obtained for PCR, the field was heavily infiltrated with CD8 + T cells, PD-1 + lymphocytes, CD68 + macrophages, FoxP3 + regulatory T cells, and tertiary lymphoid structures (TLS) (50).

Later research used RNA sequencing analysis of multiple immune cell subtypes in the tumor microenvironment to predict the efficacy of neoadjuvant immunotherapy. In the LCMC3 study (45), Ig-like transcript 2 (ILT2) was positively correlated with MPR by single-cell sequencing surgically resected specimens. ILT2 was mostly expressed in dendritic cells, monocytes, and macrophages, and was correlated with PD-L1 expression. A linear correlation suggested that LIT2 was co-expressed with PD-L1 on the same cells. The study also reported that early decreases in serum interleukin-8 (IL-8) were associated with longer overall survival (p = 0.015) (57). Low systemic inflammation, including interleukin-6 (IL-6), IL-8, and high levels of IFN-γ, was observed in patients who had a long-term response to ICI treatment (58). Transforming growth factor (TGF)-β signaling also functioned importantly in the regulation of TME. TGF-β promotes tumor invasion and metastasis by inducing the epithelial–mesenchymal transition (EMT) of NSCLC and can predict the clinical outcomes of patients with lung adenocarcinoma (LUAD) who are treated with immunotherapy (59, 60).

### 5.3 Host-associated biomarkers

Host-related biomarkers include general characteristics (such as gender, age, and body fat distribution.), gut symbionts, and host germline genetic characteristics (such as human leukocyte antigen, HLA, diversity, and other specific mutations). The immune microenvironment is different after immunotherapy. By comparing the gene expression profiles of surgically resected specimens with normal lung samples, it is possible to use the NSCLC immune microenvironment to predict surgical outcomes. Peripheral T-cell receptor sequencing may also prove useful in predicting a patient’s response to immunotherapy. An increased abundance of gut *Ruminococcus* and *Akkermansia* spp. was associated with MPR to dual therapy (16). Another study that performed a microbial analysis found that *Parabacteroides distasonis* and *Bacteroides vulgatus* abundance was higher in anti-PD-1 blockade responders than in non-responders (61).

A high neutrophil-to-lymphocyte ratio (NLR) and platelet-to-lymphocyte ratio (PLR) are indicators of host inflammation and associated with worse overall survival (OS) in NSCLC. Elevated pretreatment NLR and PLR are also associated with shorter OS and PFS and worse response rates in patients with metastatic NSCLC treated with nivolumab independent of other prognostic factors (62). Carcinoembryonic antigen (CEA) and cytokeratin-19 fragment (CYFRA 21-1), which have been used for decades to monitor the efficacy of antitumor therapy, may also be useful in predicting NSCLC patient outcome (63, 64).

### 5.4 Blood cell and liquid biopsy-related biomarkers

#### 5.4.1 Circulating tumor DNA monitoring during perioperative immunotherapy

Cell-free DNA in plasma is called circulating cell-free DNA (cfDNA). cfDNA exists in various body fluids of the human body, and its concentration changes with tissue damage, cancer, and inflammatory reactions, where cells from tumor patients are released into the body (65). Circulating cfDNA is ctDNA. Postoperative ctDNA is non-invasive mode of minimal residual disease (MRD) detection *via* liquid biopsy and has been widely used as a prognostic biomarker in patients with early NSCLC (66). A study that dynamically tracked 40 NSCLC patients with stage I–III disease after radical treatment using CAPP-Seq technology showed that all 20 ctDNA-positive patients at any time point had disease recurrence, with a median advance prediction time of 5.2 months (67, 68).

Comparing ctDNA levels before and after surgery may help identify patients at a high risk for disease recurrence. A retrospective study including 22 patients with stage IB–IIIA NSCLC who received neoadjuvant immunotherapy combined with chemotherapy, double immunotherapy, or chemotherapy alone used lung cancer-MRD sequencing panels for ctDNA detection before and after neoadjuvant therapy postoperatively and during follow-up. Patients who were ctDNA detection positive after neoadjuvant therapy and before surgery had a poorer RFS prognosis (HR, 7.41; 95% CI, 0.91–60.22, p=0.03). ctDNA was detected in 31.8% of patients 3–8 days after surgery and was found to be an independent risk factor for recurrence (HR, 5.37; 95% CI, 1.27–22.67, p=0.01). ctDNA detection 3 months after surgery suggests that it could predict recurrence, with a sensitivity and specificity of 83% and 90%, respectively (69). Another study of patients with higher stage (III/IV) disease found that those who were preoperative ctDNA positive had a significantly lower RFS (HR = 3.812, p = 0.0005) and OS (HR = 5.004, p = 0.0009), with ctDNA detection ahead of radiographic findings by a median of 12.6 months (41).

Early findings from NADIM study suggest that ctDNA clearance may be superior to radiological assessment at predicting survival and that neoadjuvant nivolumab combined with chemotherapy for resectable NSCLC could achieve a long-term survival benefit (70). CheckMate 816 also suggested that ctDNA may predict long-term DFS and OS and that higher pCR and longer EFS can be seen in patients with ctDNA clearance (14). A prospective, multicenter cohort enrolled 950 plasma samples obtained at three perioperative time points (before surgery and 3 days and 1 month after surgery) of 330 stage I–III NSCLC patients. Perioperative ctDNA analysis was found to be effective in the early detection of MRD and relapse risk stratification and hence could benefit NSCLC patient management (71).

A study reported at the 2022 ASCO Annual Meeting revealed that ctDNA detection after surgery could indicate an increased risk of recurrence risk when monitoring the effects of adjuvant therapy in patients with resectable NSCLC. The recurrence ratio was 33.33% (4/12) in patients with detectable ctDNA and 4.34% (1/23) in patients with undetectable ctDNA before adjuvant therapy. After a median follow-up of 9.47 months, 6 patients relapsed. All patients who were ctDNA negative were disease-free after adjuvant therapy (11/11), while those who were ctDNA positive had a 33.33% (1/3) recurrent rate (72). The outcomes of IMpower010 showed that adjuvant atezolizumab combined with chemotherapy had a DFS benefit in patients who were ctDNA positive. Adding adjuvant atezolizumab can reduce the risk of recurrence by 28% and in both ctDNA-positive and ctDNA-negative patients. Adjuvant Atezo plus chemotherapy was effective only in patients with positive PD-L1 expression (38). Yilong Wu et al. recently explored the prognostic value of MRD detection in patients with NSCLC after surgery, reporting a negative predictive value of 96.8%. This might represent potentially cured patients regardless of stage and adjuvant therapy (73).

Several clinical studies are under way to assess the perioperative use of ctDNA. NCT04966663 is attempting to use ctDNA detection to help in predicting if giving adjuvant treatment after surgery can decrease the chance of lung cancer recurrence, while NCT04585477 explores if the administration of durvalumab can reduce the number of circulating cancer cells in the blood after testing positive for residual cancer after standard treatment. Other studies have attempted to use ctDNA as a biomarker with different immunological agents like nivolumab (NCT03770299) and atezolizumab (NCT04267237) added to standard of care therapy (SOC) after surgery to test their effectiveness compared with SOC alone. The MEDAL study (NCT03634826) is trying to prospectively confirm the value of circulating tumor DNA and its aberrant methylation in their longitudinal monitoring of surgical lung cancer patients.

Despite the prognostic advantages of ctDNA, it is not highly sensitive (20%) and may not predict the MRD of patients with brain metastases (73). Additional studies are needed to explore the underlying mechanism behind this phenomenon.

#### 5.4.2 Peripheral blood cells and other molecular biomarkers

Additional biomarkers are related to peripheral blood cells (e.g., CD45RO+/CD8+T cells, circulating tumor cells, CTCs, and other molecular markers such as exosomes) (74). Exploration of peripheral blood immune phenotypes in the prediction of MPR and innate immune cells such as natural killer (NK) cells and NK-like T cells expressing ILT2 and NKG2A in the peripheral blood may be able to quantify the efficacy of neoadjuvant immunotherapy.

CheckMate 159 explored the relationship between the efficacy and specific expansion of tumor-specific T cells in the peripheral blood and found that tumor-specific T-cell subtypes continue to increase with treatment and continued disease-free status in MPR patients but decreased in patients with recurrent disease or who did not achieve MPR (75). In LCMC3, a lower frequency of ILT2+NKG2A+ and ILT2+NKG2A natural killer (NK) cells and ILT2+ NK-like T cells in pretreatment peripheral blood was significantly associated with MPR, suggesting that ILT2 has a negative effect on the HLA-G and/or NKG2A/HLA-E axis. NKG2D expression on NK cells correlated with lymph node involvement, whereas expression on NK-like T cells and T cells correlated with no lymph node involvement, suggesting that the NKG2D/NKG2D-L axis plays a role in tumor immune escape. These immunophenotypic data identify new potential immune escape mechanisms and new potential biomarkers and therapeutic targets (45).

## 6 Discussion

Current clinical research findings suggest that the use of a PD-1 inhibitor combined with chemotherapy (neoadjuvant chemotherapy immunotherapy) is superior to neoadjuvant PD-1 monotherapy and dual immunotherapy (76).

During the perioperative period, treatment strategies should be classified according to their precise modality. Accurate TNM staging is beneficial to identifying the appropriate treatment strategy. The pathological classification of tumors can help guide the scope of surgical resection. Qiu presented at the 2022 ASCO meeting that the MPR rate of squamous cell carcinoma patients (51.6%, 16/31) was significantly higher than those with non-squamous cell carcinoma (12.5%, 3/24) (p = 0.002) (77). Furthermore, the tumor microenvironment can predict tumor evolution and development, while molecular sub-types represent their heterogeneity. The endpoints assessed for neoadjuvant and perioperative therapy were EFS, pCR, and MPR, while those for adjuvant therapy were DFS and OS.

Lung cancer postoperative recurrence and metastasis is still a major problem. The more advanced the stage, the higher the risk of recurrence and metastasis (14).

The 5-year survival rate of stage I patients was 90% after surgical treatment, with similar local and distant recurrence rates of approximately 5% each. In contrast, the local recurrence rate of stage IIB–IIIA patients is 12%–15%, with a distant recurrence rate of 40%–60% (15). Adjuvant therapy is therefore recommended for perioperative patients with stage II–IIIA disease and may also be recommended for select stage IB patients as well. Although important clinical trials are still ongoing, already completed trials have yielded exciting preliminary results for immunotherapy, with an MPR of 22%–45% for immune monotherapy and 50%–83% for immunotherapy combined with chemotherapy (15–17, 31). The safety of immunotherapy is good, indicating that neoadjuvant immunotherapy is a promising treatment strategy for patients with resectable lung cancers. Compared with the adjuvant approach, neoadjuvant therapy can help eliminate micrometastases early on. However, despite effective treatment, the study arm was terminated early due to toxicity (50).

Surgical safety after neoadjuvant immunotherapy is of interest to surgeons, but there are no current indicators that immunotherapy impact surgical outcomes. A prior work showed that neoadjuvant immunotherapy alone or in combination with chemotherapy did not result in many delayed surgical events, with an overall mean surgical resection rate of 88.7%. There was also no increase in surgical difficulty and perioperative risk. The mean incidence of surgical complications was 20.6%, with most patients having good prognosis. Deaths had almost nothing to do with drug treatment (30).

Immune single-agent neoadjuvant studies, such as CheckMate 159, LCMC3, PRINCEPS, TOPlS01, IoNESCO, and ChicTR-OIC-17013726, included patients with stage I–IIIb NSCLC. After one to three cycles of treatment, MPR ranged from 14% to 45%. Neoadjuvant immune combined chemotherapy (NADIM, NCT02716038, SAKK 16/14) studies enrolled patients with stage Ib–IIIa disease for two to four cycles. The MPR rate of the NADIM study was as high as 85.36%, and pCR reached 71.4%. The MPR of both the other studies was approximately 60%. The double immune neoadjuvant study (NEOSTAR) enrolled patients with stage I–IIIA lung cancer for three cycles of treatment, yielding an MPR rate of 24% (16). CheckMate 816 is currently the first phase III study to reach its primary endpoint, and we are eagerly awaiting the release of more detailed data (14).

In order to explore correlations between pathological endpoints and long-term benefit and as more trials use MPR and pCR as surrogates for the clinical benefit of neoadjuvant therapy, there is an urgent need to clearly demonstrate to what extent these pathological endpoints reflect survival benefit. A retrospective analysis showed that patients who achieved MPR after neoadjuvant chemotherapy had significantly prolonged DFS and OS compared with those who did not. Thus, does this effect apply to immune combination therapy? In NADIM (nivolumab plus chemotherapy), the 24-month OS was 100% in patients who achieved MPR or pCR and 85.7% in patients with an incomplete pathological response (p=0.002). Another clinical trial of neoadjuvant immunotherapy plus chemotherapy (atezolizumab combined with chemotherapy) reported that the median DFS of patients who achieved versus who did not achieve MPR was 34.5 and 14.3 months, respectively (p=0.71) (25). The relationship between pCR, MPR, and OS still requires a large amount of clinical data to verify, and the definition of MPR needs to be further explored and standardized.

Delayed surgery may lead to tumor progression. Reasons why surgery may be delayed include differences in imaging judgment and treatment-related adverse reactions. Premature surgery may cause serious surgical complications in addition to the immune cell infiltration stimulated by immunotherapy during the anti-tumor response. It is therefore necessary to determine the optimal time interval between neoadjuvant immunotherapy and surgery. Too long or too short of an interval will reduce the efficacy of immunotherapy. Meanwhile, preclinical research indicates that changes in the immune microenvironment may assist with surgical timing, and the effects on surgery caused by prolonging the interval may be related to the proportion of tumor-specific T cells and IFNγ production (42).

It is still difficult to screen for immune responders using PD-L1, TMB, or other kinds of tumor immune microenvironment markers. Assessment of the pathological response using both the primary tumor and lymph nodes (LNs) may be important ways to judge the efficacy of neoadjuvant immunotherapy (78). Furthermore, is it important to question if the supplementation of MPR and pCR with surrogate or complementary indicators is the right way to monitor postoperative outcomes over the long term? Moreover, what are common microenvironmental changes that occur after immunotherapy? Available data from CheckMate816 showed a tumor PD-L1 expression level more than 1% and stage III resectable NSCLC are predictors of outcome (14). Furthermore, the sensitivity of single postoperative ctDNA MRD detection is <50%. Although continuous longitudinal ctDNA MRD detection can greatly improve its sensitivity, its sensitivity among patients with brain metastases is not high (20%) and MRD may not be detected (73). More clinical studies are needed to validate the predictive merits of any of these factors.

Current perioperative immunotherapy research study designs are not the same. Many issues such as the selection of neoadjuvant therapy, the use of a postoperative immune adjuvant, the duration of adjuvant therapy, the selection of the target population, and postoperative recurrence and metastasis monitoring still require further study. New long-term survival follow-up data are being released. The 5-year clinical outcome of neoadjuvant therapy with nivolumab was better than that of historical controls, with a 5-year OS of 80%, a 5-year RFS of 60%, and a median RFS of 67 months (46). The “International Expert Consensus on Neoadjuvant Immunotherapy for Non-Small Cell Lung Cancer” (27), the “Expert Consensus on Perioperative Immunotherapy for Locally Advanced Non-Small Cell Lung Cancer” (28), and the “Consensus on Postoperative Recurrence Prediction of Non-small Cell Lung Cancer Based on Molecular Markers” (29) have been published. We look forward to more results of phase III studies on neoadjuvant/adjuvant immunotherapy for NSCLC.

**Figure 2:**
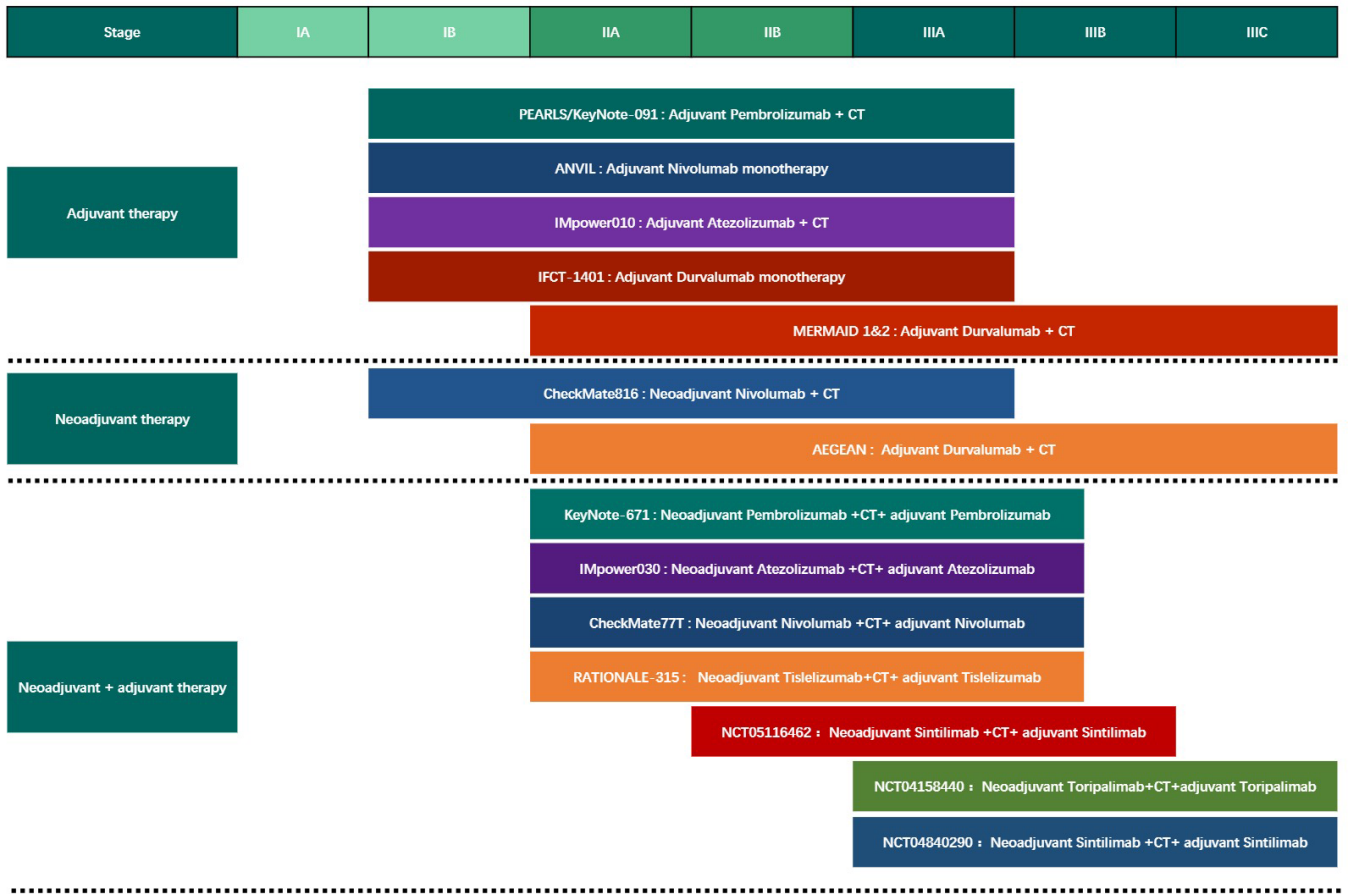
The Phase III clinical trials of different modes of perioperative immunotherapy for Non-Small Cell Lung Cancer. CT, chemotherapy.

Compared with advanced tumors, perioperative treatment research over a large time span encompasses many significant changes during the treatment period. Many factors such as treatment drugs, treatment strategies, and population distribution may change dramatically, affecting research progress and results. A larger volume of research is needed to combat these potential biases (**Figure 2**).

## 7 Conclusion

In conclusion, perioperative neoadjuvant and adjuvant ICI monotherapy and immune-combination therapy have successfully improved the survival and prognosis of patients with resectable NSCLC. Recent clinical trial findings suggest that patients who receive neoadjuvant and adjuvant immunotherapy combined with chemotherapy had better outcomes and acceptable safety than other modalities. Perioperative immune-related drugs and interval cycles varied between studies, with most studies choosing two to four cycles to ensure its efficacy and patient compliance. More clinical evidence is required to identify the optimal regimen. Different tumor biomarkers such as TME, host associated, blood cell, and liquid biopsy were also evaluated. Additional studies and clinical research findings are needed to identify the ideal perioperative regimen.

## Author contributions

FW: Conceptualization, Supervision, Validation, Design the figure. YRP, ZL: Writing original draft, Design the figure and tables. YF: Drawing the figure. YP, YZ, JL, CX, YZZ, YS, GL: Review, Editing. All authors contributed to the article and approved the submitted version.
