# Corrigendum: Progress and perspectives of perioperative immunotherapy in non-small cell lung cancer

**DOI:** 10.3389/fonc.2023.1163376

**Published:** 2023-03-29

**Authors:** Yurong Peng, Zhuo Li, Yucheng Fu, Yue Pan, Yue Zeng, Junqi Liu, Chaoyue Xiao, Yingzhe Zhang, Yahui Su, Guoqing Li, Fang Wu

**Affiliations:** ^1^ Department of Oncology, the Second Xiangya Hospital, Central South University, Changsha, Hunan, China; ^2^ The Ophthalmologic Center of the Second Xiangya Hospital, Central South University, Changsha, Hunan, China; ^3^ XiangYa School of Public Health, Central South University, Changsha, Hunan, China

**Keywords:** perioperative period perioperative immunotherapy, neoadjuvant therapies, immunotherapy, neoadjuvant immune monotherapy, adjuvant therapy, biomarkers, NSCLC, lung cancer

In the published article, two figures (**1** and **2**) were accidentally omitted but were subject to peer review and included in the original submission. The figures and respective citations have been added to the original article. **Figure 1** has been cited at the end of **1 Introduction** and **Figure 2** has been cited at the end of the **6 Discussion**. The missing figure and their captions appear below.

**Figure 1:**
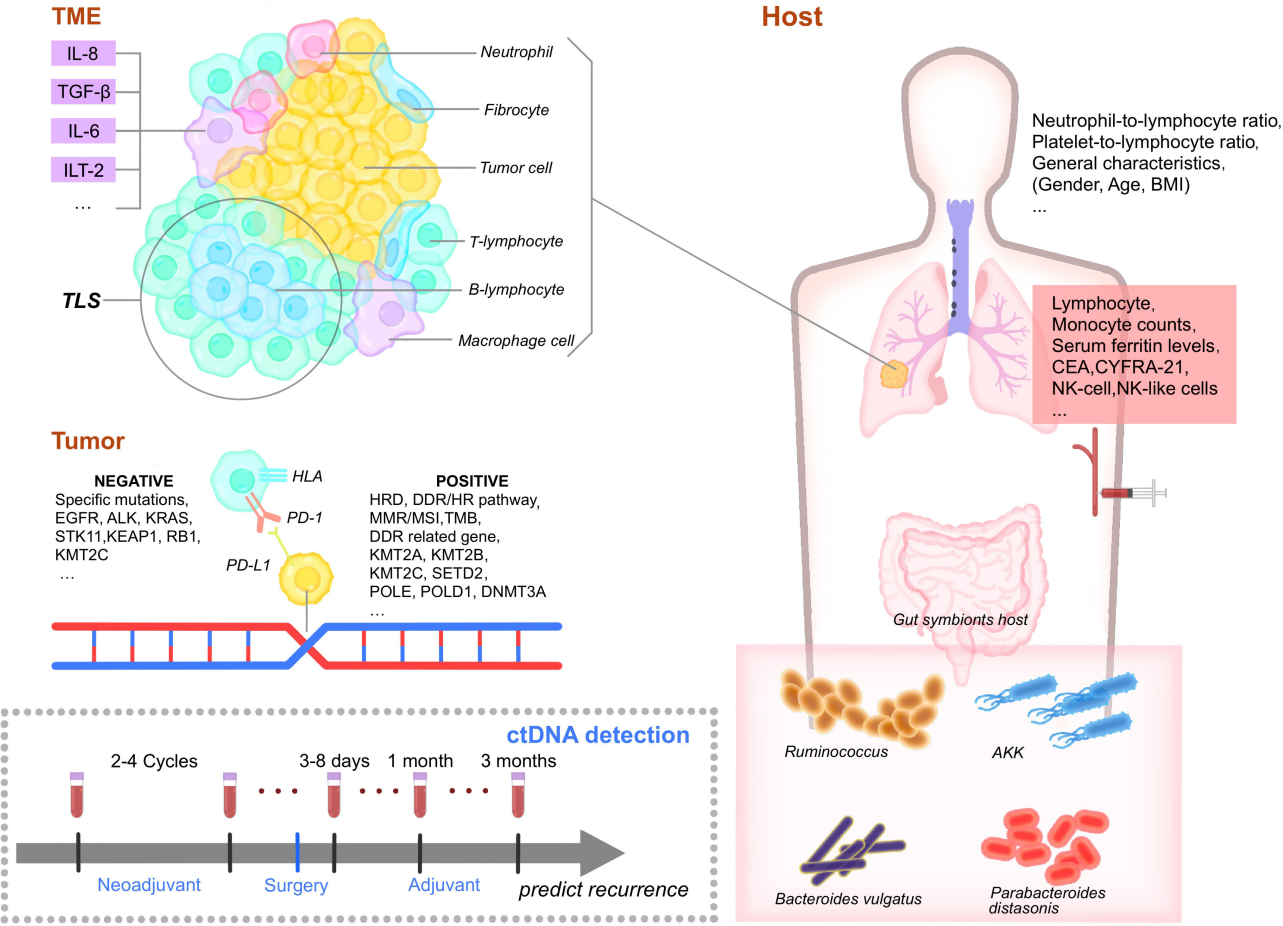
Biomarkers of perioperative immunotherapy. TME, Tumor microenvironment. IL-8, interleukin-8. TGF-β, Transforming growth factor. IL-6, interleukin-6. ILT-2, Ig-like transcript 2. TLS, Tertiary lymphoid structures. TIL, tumor-infiltrating lymphocytes. EGFR, epidermal growth factor receptor. HLA, human leukocyte antigen. PD-1, programmed cell death protein 1. PD-L1, Programmed cell death ligand 1. HRD, Homologous recombination deficiency. DDR, DNA-damage response/HR, homologous recombination pathway, MMR, mismatch repair. MSI, microsatellite instability. TMB, tumor mutation burden. KMT2A/B/C, Lysine methyltransferase 2A/B/C. POLE, polymerase epsilon. DNMT3A, DNA methyltransferases 3A. BMI, body mass index. CEA, carcinoembryonic antigen. NK, natural killer cells. AKK, Akkermansia muciniphila. NK, natural killer cells. CD4, cluster of differentiation 4. CD8+, cluster of differentiation 8. Treg, regulatory T cells. DC, dendritic cells. CTL, cytotoxic T lymphocyte.

**Figure 2:**
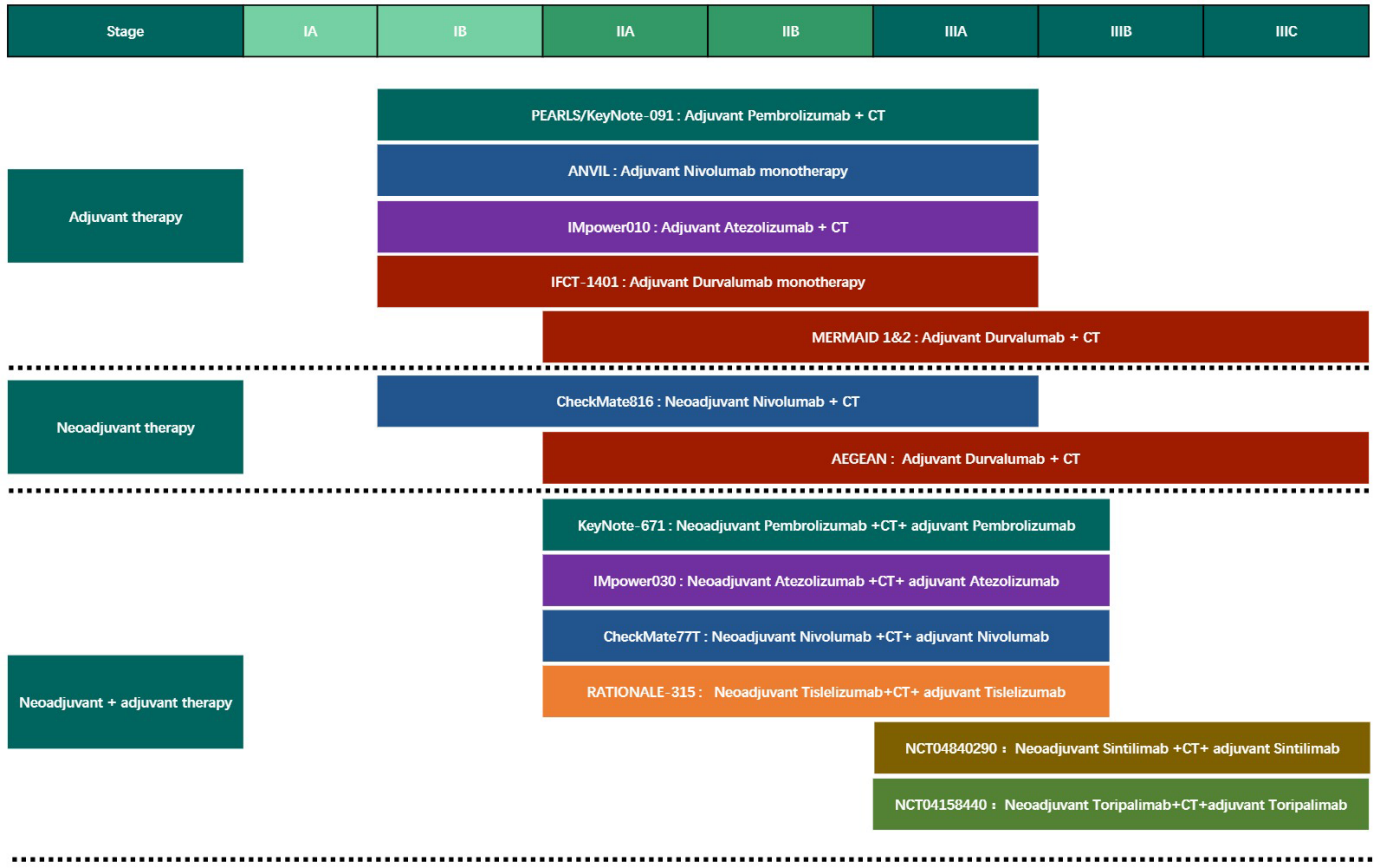
The Phase III clinical trials of different modes of perioperative immunotherapy for Non-Small Cell Lung Cancer. CT, chemotherapy.

The authors apologize for this error and state that this does not change the scientific conclusions of the article in any way. The original article has been updated.

